# Magnetic Nanoparticles for Drug Delivery through Tapered Stenosed Artery with Blood Based Non-Newtonian Fluid

**DOI:** 10.3390/ph15111352

**Published:** 2022-11-01

**Authors:** Muhammad Mubashir Bhatti, Sadiq M. Sait, Rahmat Ellahi

**Affiliations:** 1 College of Mathematics and Systems Science, Shandong University of Science and Technology, Qingdao 266590, China; 2 Center for Communications and IT Research, Research Institute, King Fahd University of Petroleum & Minerals, Dhahran 31261, Saudi Arabia; 3 Center for Modeling & Computer Simulation, Research Institute, King Fahd University of Petroleum & Minerals, Dhahran 31261, Saudi Arabia; 4 Department of Mathematics & Statistics, Faculty of Basic and Applied Sciences, International Islamic University, Islamabad 44000, Pakistan; 5 Fulbright Fellow Department of Mechanical Engineering, University of California Riverside, Riverside, CA 92521, USA

**Keywords:** magnetic drug delivery, hybrid nanofluid, magnetic field, non-Newtonian fluid, heat transfer

## Abstract

Nanoparticles play an essential role in biomedical applications. A most promising area in nanomedicine is drug targeting which is done with the aid of magnetized nanoparticles. In this study, the hemodynamics of hybrid nanofluid flow with gold and copper nanoparticles suspended in it is investigated. This research primarily focuses on magnetic drug delivery which is propagated through a tapered stenosed artery under three situations, including converging, diverging, and non-tapering arteries. To explore the rheological characteristics of blood, a Sutterby fluid, which is a non-Newtonian fluid, is postulated. The energy equation also incorporates the effects of the magnetic field and joule heating, as well as the viscous dissipation function. Lubrication theory provides a mathematical framework for model formulation. The hypothesized modeling is simplified to a set of nonlinear differential equations that are then solved using a perturbation method up to the second order of approximation. Graphs are used to describe the outcomes of different evolving parameters. The Sutterby fluid parameter opposes the flow negligibly, whereas the Hartmann number and thermal Grashof number strengthen the flow field. Copper nanoparticles (in the absence of gold nanoparticles) are observed to deplete the thermal profile substantially more than gold nanoparticles. Nevertheless, the thermal profile is enhanced by the presence of both nanoparticles (hybrid nanofluids). For greater values of the Sutterby fluid parameter, the wall shear stress has been observed to rise considerably, whereas the inverse is true for the Hartmann number and the thermal Grashof number. The present results have been improved to give significant information for biomedical scientists who are striving to study blood flow in stenosis situations, as well as for those who will find the knowledge valuable in the treatment of different diseases.

## 1. Introduction

Nanotechnology is a significant issue in contemporary science. Scientists, chemists, medics, and engineers may use nanotechnology to operate on cellular and molecular levels, bringing considerably advanced technology to health care and life sciences. The word nano is derived from the Greek word nanos, which means small. According to the ASTM (American Society for Testing and Materials); an international 2006 report, nanoparticles are particles with two or more dimensions and a size ranging from 1–100 nm [1]. Due to its unique size and physiochemical properties, the use of nanoparticles offers numerous advantages. Due to their enormous reactive and exposed surface area and quantum size influence, these particles offer superior chemical and physical properties compared to bulk materials. Nanoparticles have found extensive use in a multitude of areas, including biology, chemistry, photochemistry, and electronics [2].

The existence of an external magnetic field on nanoparticles is also significant in biological research, material science, biotechnology, engineering, and environmental sciences [3,4,5]. Magnetic nanoparticles in biomedical engineering are further classified into two types based on their application, such as in vivo and in vitro [6,7,8]. In vivo applications included diagnostic (nuclear resonance imaging) and therapeutic (drug-targeting and hyperthermia), while in vitro applications included magneto-relaxometry, selection, and diagnostic separation. Different physical qualities are required for each conceivable use of magnetic nanoparticles. Magnetized nanoparticles exhibit spectacular new phenomena such as a high saturation field, increased anisotropy contributions, shifted loops, high field irreversibility, and super-para-magnetism. These events arise as a result of limited and narrow size impact and surface impact, which determine each particle’s magnetic behavior [9]. Nanoparticle-based drug delivery systems have been shown to be the most effective way of treating brain cancers when traditional treatment is ineffective. The nanoparticles’ enhanced permeability and retention are the distinguishing characteristics that allow them to aggregate and engage with tumor cells [10]. Platelets have a key role in the generation of cardiovascular diseases, hence antiplatelet therapy is thought to be an essential component in the treatment of such diseases. As a result, the interaction between nanoparticles with the circulatory system becomes a significant aspect of the prevention and treatment of cardiovascular disease. The main qualities of magnetic nanoparticles that make them an effective drug delivery tool are cytotoxicity, a simple and direct method of contact with desired target, and mono-dispersity. Various types of nanoparticles have been produced based on these properties for medication delivery and imaging i.e., copper, quantum dots, gold nanoparticles, silver nanoparticles, dendrimers, iron oxide, and zinc oxide nanoparticles [11].

When compared to other types of metallic nanoparticles, gold and copper nanoparticles were shown to be the least harmful and most effective agents for the transportation of drugs and for hyperthermia agents [12]. Due to their effective and precise medication delivery to the tumor sites, gold nanoparticles have been used in the fields of radiation enhancement and radiation medicine [13]. They also exhibit higher therapeutic in radiation treatment. The biocompatibility of nanoprobes based on gold nanoparticles is intriguing for molecular imaging of numerous metabolites and enzymes that are essential for cancer cell activity. Gold nanoparticles provide many benefits over typical iodine-based treatments. For example, they have a greater absorption coefficient than iodine because of their higher electron density and atomic number [14]. Second, gold nanoparticles are non-cytotoxic, and third, they have a greater surface area, which allows for surface modification with targeting molecules [15]. Copper nanoparticles have also piqued the interest of researchers owing to their many implications in medicine, lubricants, optics, antibacterial agents, conductive files, nanofluids, and electronics [16,17,18,19,20]. Copper nanoparticles are less expensive, have high chemical and physical stability, and can be conveniently combined with polymers [21].

In light of the extensive uses of nanofluids in diverse sectors, several authors have investigated their behavior under a variety of conditions and geometrical shapes. For instance, Ardahaie et al. [22] analyzed the mechanism of nanofluid using a porous artery with an extrinsic magnetic field. They discovered that enhancing the adverse pressure gradient with the thermophoresis parameter increases blood flow, while a magnetic field diminishes blood flow. Ellahi et al. [23] addressed peristaltic induced blood flow computationally using a non-Newtonian chemicalized couple stress nanofluids with activation energy. Elmaboud et al. [24] used a computational model of gold nanoparticles with peristaltic dynamics and a magnetic field to evaluate blood rheology. They used a couple stress fluid model to explore blood rheology. Seikh et al. [25] analyzed slip effects and magnetic field dynamics on blood flow across arterial veins. They discovered, using analytical solutions, that viscous dissipation optimizes the thermal profile while the velocity field drops owing to the substantial influence of the magnetic field. Mekheimer et al. [26] addressed the phenomenon of gold nanoparticles passing through a stenosed tapering artery under electrothermal impact. They discovered that gold nanoparticles improve heat transfer in fluids, which will benefit in thermal therapy. Zhang et al. [27] analyzed the performance of entropy formation on blood circulation using magnetized ZnO nanoparticles in a tapered artery. They developed a mathematical mode and showed the specific analytical results using the Jeffrey fluid model and lubrication technique. Umadevi et al. [28] explored blood rheology in the existence of a magnetic field on an oblique stenosed artery adopting copper nanoparticles. Few noteworthy relevant studies on the topic under investigation can be found in the references [29,30,31,32,33,34,35,36,37,38,39,40,41,42,43] and several others.

Following the detailed analysis of the literature, it was discovered that little focus was given to the blood flow under the suspension of copper and gold nanoparticles, with a particular focus on magnetic drug delivery. Magnetic nanoparticles have a vital role in the treatment of a variety of disorders, including cancer. The proposed nanoparticles are also helpful to treat diabetic patients. Drug targeting has attracted a lot of attention in recent years as one of the most developed technologies. Magnetized nanoparticles, in conjunction with an extrinsic magnetic field and magnetized implants, allow particles to be delivered to the precisely desired spot, fixed at the local site while the medicine is released, and act locally. Transporting drugs to a designated place may eliminate adverse effects and reduce the necessary dose. Gold and copper nanoparticles have excellent bio-compatibility and economic viability; therefore, it has multitudinous engineering applications in medical science. A catheterized tapered artery is considered, which is filled with non-Newtonian Sutterby fluid with copper and gold nanoparticles under the presence of the extrinsic magnetic field. Lubrication theory and perturbation approaches are used to obtain the solutions for the nonlinear differential equations. The results are discussed with the help of graphs for all the emerging parameters. The obtained findings will be helpful for the experimental work on the heat transfer with Sutterby magnetized hybrid nanofluid flows. The present results have been improved to give significant information for biomedical scientists who are striving to study blood flow in stenosis situations, as well as for those who will find the knowledge valuable in the treatment of different diseases.

## 2. Materials

**Figure 1:**
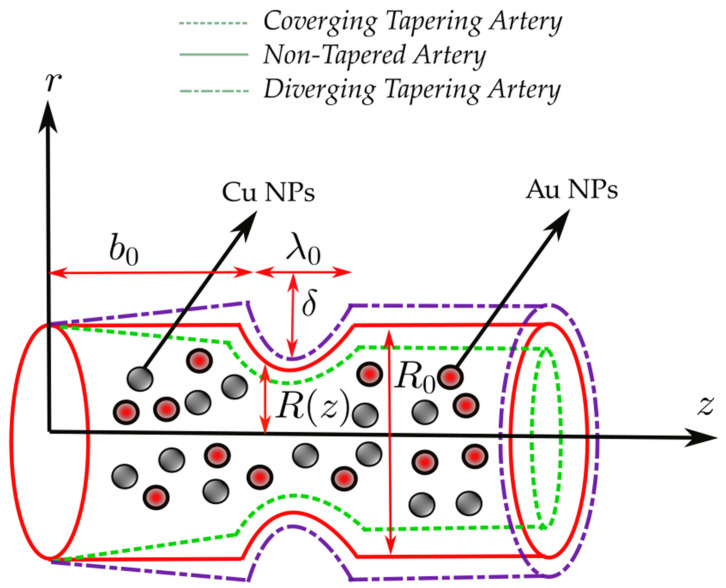
Geometrical structure of the blood flow through a stenosed tapered artery filled with Au-Cu Nanoparticles.

Consider a catheterized tapered artery having length $\lambda_{0}$ filled with hybrid Sutterby nanofluid in the presence of Gold (Au) and Copper (Cu) nanoparticles. The Sutterby nanofluid is considered as blood with incompressible and electrically conducting features. An intense external magnetic field causes the Sutterby nanofluid to be electrically conductive. The system of cylindrical polar coordinates (r,θ,z) is considered, whereas *r* is located in the radial direction, *z* is located along the flow direction and θ is located along the circumferential direction. Furthermore, the third-grade nanofluid propagates in the presence of heat transfer phenomena where ${\tilde{T}}_{1}$ is the temperature at the wall. The mathematical form of the proposed geometry is described as:

(1) $$R(z)=\left\{ \begin{array}{l} \left(R_{0}+z\bar{\xi }\right)\left[1-\frac{n^{\frac{n}{n-1}}\delta }{\lambda_{0}^{n}R_{0}\left(n-1\right)}\left\{\lambda_{0}^{n-1}\left(z-b_{0}\right)-{\left(z-b_{0}\right)}^{n}\right\}\right],\;b_{0}<z\leq \lambda_{0}+b_{0},\\ \left(R_{0}+z\bar{\xi }\right)\;\;\;\;\;\;\;\;\;\;\;\;\;\;\;\;\mathrm{otherwise} \end{array}\right.$$

where δ represents the height of the stenosis which is situated at $z=b_{0}+\frac{\lambda_{0}}{n^{\left(n-1\right)/n}}$, $R_{0}$ is the radius of the non-tapered artery in a non-stenotic part, $\bar{\xi }=\tan\psi$ is the tapering parameter, i.e., ψ is the tapering angle where the non-tapered artery is at ψ=0, diverging tapered artery is at ψ>0 and converging tapered artery is at ψ<0. Also, $\lambda_{0}$ is the length of the stenosis, n≥2 represents the configuration of the constriction profile which belongs to the shape parameter for which symmetric stenosis can be obtained for n=2, and $b_{0}$ shows the location of the stenosis as displayed in the Figure 1.

The equations governing the hybrid nanofluid flow can then be expressed as below [44]:

(2) $$\nabla \cdot \tilde{V}=0,$$

(3) $$\rho_{hnf}\left(\frac{\partial \tilde{V}}{\partial t}+\tilde{V}\cdot \nabla \tilde{V}\right)=-\nabla \cdot p+\nabla \cdot \zeta +J\times B+g{\left(\rho \tilde{\beta }\right)}_{hnf}\left(\tilde{T}-{\tilde{T}}_{1}\right),$$

where $\tilde{V}$ has the components of velocity, B is the magnetic field, J is the current density, $J\times B=-\Omega_{hnf}B_{0}^{2}\tilde{V}$, $\Omega_{hnf}$ is the electrical conductivity of hybrid nanofluid, $B_{0}$ is the applied magnetic field, $\tilde{\beta }$ denotes the coefficient of the thermal expansion, $\tilde{T}$ is the nanofluid temperature, g is the acceleration due to gravity, p is the pressure, $\rho_{hnf}$ represents the density of hybrid nanofluid, $\mu_{hnf}$ is the viscosity of the hybrid nanofluid, hnf in subscript represents the hybrid nanofluid and ζ is the stress tensor for the *Sutterby fluid* model which is defined below [45]:

(4) $$\zeta =\mu_{hnf}{\left[\frac{\sinh^{-1}\left(B\dot{\Pi }\right)}{B\dot{\Pi }}\right]}^{N}R_{1},\dot{\Pi }={\left[\frac{\mathrm{trac}\left(R_{1}^{2}\right)}{2}\right]}^{1/2},R_{1}=\mathrm{grad}\tilde{V}+{\left(\mathrm{grad}\tilde{V}\right)}^{T},$$

where *B* is the material constant, $R_{1}$ is the first Rivlin-Erickson tensor.

The temperature equation with viscous dissipation and joule heating functions reads as follows:

(5) $${\left(\rho C_{p}\right)}_{hnf}\left(\frac{\partial \tilde{T}}{\partial t}+\tilde{V}\cdot \nabla \tilde{T}\right)=\nabla \cdot \left(k_{hnf}\nabla \tilde{T}\right)+\frac{J\cdot J}{\Omega_{hnf}}+\zeta \cdot \nabla \tilde{V},$$

where $\kappa_{hnf}$ and ${\left(\rho C_{p}\right)}_{hnf}$ characterize the thermal conductivity and the heat capacity of the hybrid nanofluid, respectively.

Table 1 shows the thermo-physical attributes of density, dynamic viscosity, heat capacity, thermal conductivity, thermal expansion coefficient, and electric conductivity.

Using the lubrication technique, the hypothesized governing equation may be modeled. To achieve the suggested model in dimensionless form, the scaled variables are thus specified as follows:

(6) $$\begin{array}{l} \tilde{r}=\frac{r}{R_{0}},\tau =\frac{\lambda_{0}\tau }{R_{0}},\tilde{v}=\frac{v\lambda_{0}}{u_{\mathrm{ave}}\delta },\tilde{R}=\frac{R}{R_{0}},\tilde{p}=\frac{R_{0}^{2}}{u_{\mathrm{ave}}\lambda_{0}\mu }p,\\ \tilde{z}=\frac{z}{R_{0}},T=\frac{\tilde{T}-{\tilde{T}}_{1}}{{\tilde{T}}_{0}-{\tilde{T}}_{1}},\tilde{\delta }=\frac{\delta }{R_{0}},\lambda =\frac{L}{\lambda_{0}},\tilde{w}=\frac{w}{u_{\mathrm{ave}}}. \end{array}|$$

We construct the following set of nonlinear couple differential equations with respective corresponding boundary conditions by substituting the aforementioned Equation (6) into the governing equations (ignoring the ~ sign):

(7) $$\begin{array}{l} \frac{\partial p}{\partial z}=\frac{E_{1}}{r}\frac{\partial }{\partial r}\left[r\left\{\frac{\partial w}{\partial r}-\beta {\left(\frac{\partial w}{\partial r}\right)}^{3}\right\}\right]-E_{2}\xi^{2}w+\gamma E_{3}\theta ,\\ \frac{E_{4}}{r}\frac{\partial }{\partial r}\left(r\frac{\partial \theta }{\partial r}\right)+E_{1}\beta_{m}\left[{\left(\frac{\partial w}{\partial r}\right)}^{2}-\beta {\left(\frac{\partial w}{\partial r}\right)}^{4}\right]+\beta_{m}E_{2}\xi^{2}w^{2}, \end{array}|$$

And boundary conditions are

(8) $${\frac{\partial w}{\partial r}=\frac{\partial \theta }{\partial r}=0|}_{r=0},\;\;{w=0,\;\;\theta =0|}_{r=R}.$$

And in Equation (7), β the fluid parameter, ξ the Hartmann number, $\beta_{m}$ the Brinkman number, γ the thermal Grashof number. Mathematically, they are presented as:

(9) $$\beta =\frac{NBu_{\mathrm{ave}}^{2}}{6R_{0}^{2}},\;\xi =\sqrt{\frac{\Omega_{f}}{\mu_{f}}}B_{0}R_{0},\;\beta_{m}=\frac{\mu_{f}u_{\mathrm{ave}}^{2}}{k_{f}\left({\tilde{T}}_{1}-{\tilde{T}}_{0}\right)},\;\gamma =\frac{\left({\tilde{T}}_{1}-{\tilde{T}}_{0}\right){\tilde{\beta }}_{f}gR_{0}^{3}}{\upsilon_{f}^{2}}.$$

And the remaining expression are defined as:

(10) $$E_{1}=\frac{\mu_{hnf}}{\mu_{f}},\;E_{2}=\frac{\Omega_{hnf}}{\Omega_{f}},\;E_{3}=\frac{{\left(\rho \tilde{\beta }\right)}_{hnf}}{{\left(\rho \tilde{\beta }\right)}_{f}},\;E_{4}=\frac{k_{hnf}}{k_{f}},$$

## 3. Methods and Results

To obtain the solutions of Equation (7), we will employ homotopy perturbation method [46]. This approach, which employs higher order power series solutions, is exceedingly precise and extraordinarily rapid at converging when compared to other perturbation methods [47]. It’s been used to solve a variety of nonlinear non-Newtonian fluid dynamics problems [48,49,50]. Therefore, the homotopy for Equation (7) are defined as [51]:

(11) $$h\left(\bar{u},\varepsilon \right)=\left(1-\varepsilon \right)\left[\ddot{L}\left(\bar{u}\right)-\ddot{L}\left(\dot{u}\right)\right]+\varepsilon \left[\ddot{L}\left(\bar{u}\right)-\frac{\beta }{r}{\left(\frac{\partial \bar{u}}{\partial r}\right)}^{3}-\beta \frac{\partial }{\partial r}{\left(\frac{\partial \bar{u}}{\partial r}\right)}^{3}-\frac{1}{E_{1}}\frac{dp}{dz}-\frac{E_{2}}{E_{1}}\xi^{2}\bar{u}+\frac{\gamma }{E_{1}}E_{3}\bar{\theta }\right],$$

(12) $$h\left(\bar{\theta },\varepsilon \right)=\left(1-\varepsilon \right)\left[\ddot{L}\left(\bar{\theta }\right)-\ddot{L}\left(\dot{\theta }\right)\right]+\varepsilon \left[\ddot{L}\left(\bar{\theta }\right)+\frac{\beta_{m}E_{1}}{E_{4}}\left[{\left(\frac{\partial \bar{u}}{\partial r}\right)}^{2}-\beta {\left(\frac{\partial \bar{u}}{\partial r}\right)}^{4}\right]+\beta_{m}\frac{E_{2}}{E_{4}}\xi^{2}{\bar{u}}^{2}\right],$$

where ε∈[0,1] represents the artificial parameter. The following linear operator and the initial guesses have been selected for further process:

(13) $$\begin{array}{l} \ddot{L}=\frac{1}{r}\frac{\partial }{\partial r}\left(r\frac{\partial }{\partial r}\right),\\ \dot{u}=\dot{\theta }=\frac{r^{2}-R^{2}}{4}, \end{array}|$$

Defining the expansion

(14) $$\bar{u}={\bar{u}}_{0}+\varepsilon {\bar{u}}_{1}+\varepsilon {\bar{u}}_{2}+\ldots ,$$

(15) $$\bar{\theta }={\bar{\theta }}_{0}+\varepsilon {\bar{\theta }}_{1}+\varepsilon {\bar{\theta }}_{2}+\ldots ,$$

### 3.1. Zeroth Order System

The following set of differential equations is what we get at zeroth order:

(16) $$\ddot{L}\left(\bar{u}\right)-\ddot{L}\left({\dot{u}}_{0}\right)=0,$$

(17) $$\ddot{L}\left(\bar{\theta }\right)-\ddot{L}\left({\dot{\theta }}_{0}\right)=0,$$

The solutions of the above both equations can be written as

(18) $${\bar{u}}_{0}={\bar{\theta }}_{0}=\frac{r^{2}-R^{2}}{4}.$$

### 3.2. First Order System

We acquire the subsequent set at first order:

(19) $$\ddot{L}\left({\bar{u}}_{1}\right)+\ddot{L}\left(\dot{u}\right)-\beta \frac{\partial }{\partial r}{\left(\frac{\partial {\bar{u}}_{0}}{\partial r}\right)}^{3}-\frac{\beta }{r}{\left(\frac{\partial {\bar{u}}_{0}}{\partial r}\right)}^{3}-\frac{1}{E_{1}}\frac{dp}{dz}-\frac{E_{2}}{E_{1}}\xi^{2}{\bar{u}}_{0}+\frac{\gamma }{E_{1}}E_{3}{\bar{\theta }}_{0},$$

(20) $$\ddot{L}\left({\bar{\theta }}_{1}\right)+\ddot{L}\left(\dot{\theta }\right)+\frac{\beta_{m}E_{1}}{E_{4}}\left[{\left(\frac{\partial {\bar{u}}_{0}}{\partial r}\right)}^{2}-\beta {\left(\frac{\partial {\bar{u}}_{0}}{\partial r}\right)}^{4}\right]+\beta_{m}\frac{E_{2}}{E_{4}}\xi^{2}{\bar{u}}_{0}{}^{2},$$

The solutions of the above both equations can be found as

(21) $${\bar{u}}_{1}=\frac{\left(r^{2}-R^{2}\right)\left[16dp/dz-\left(E_{3}\gamma -E_{2}\xi^{2}\right)\left(r^{2}-3R^{2}\right)+2E_{1}\left(-8+\left(r^{2}+R^{2}\right)\beta \right)\right]}{64E_{1}},$$

(22) $${\bar{\theta }}_{1}=\frac{-\left(r^{2}-R^{2}\right)\left[288E_{4}+\beta_{m}\left\{18E_{1}\left(r^{2}+R^{2}\right)+E_{2}\xi^{2}\left(2r^{4}-7r^{2}R^{2}+11R^{4}\right)\right\}\right]+2\beta_{m}E_{1}\left(r^{6}-R^{6}\right)\beta }{1152E_{4}},$$

### 3.3. Second Order System

We present the following set of differential equations of second order:

(23) $$\ddot{L}\left({\bar{u}}_{2}\right)-\frac{3\beta }{r}{\left(\frac{\partial {\bar{u}}_{0}}{\partial r}\right)}^{2}\frac{\partial {\bar{u}}_{1}}{\partial r}-\beta \frac{\partial }{\partial r}\left[3{\left(\frac{\partial {\bar{u}}_{0}}{\partial r}\right)}^{2}\frac{\partial {\bar{u}}_{1}}{\partial r}\right]-\frac{E_{2}}{E_{1}}\xi^{2}{\bar{u}}_{1}+\frac{\gamma }{E_{1}}E_{3}{\bar{\theta }}_{1},$$

(24) $$\ddot{L}\left({\bar{\theta }}_{1}\right)+\frac{\beta_{m}E_{1}}{E_{4}}\left[2\frac{\partial {\bar{u}}_{0}}{\partial r}\frac{\partial {\bar{u}}_{1}}{\partial r}-4\beta {\left(\frac{\partial {\bar{u}}_{0}}{\partial r}\right)}^{3}\frac{\partial {\bar{u}}_{1}}{\partial r}\right]+2\beta_{m}\frac{E_{2}}{E_{4}}\xi^{2}{\bar{u}}_{0}{\bar{u}}_{1},$$

The solutions of the above both equations can be found as

(25) $$\begin{array}{ll} {\bar{u}}_{2} & =\frac{\left(r^{2}-R^{2}\right)}{36864E_{1}^{2}E_{4}}\times 16E_{2}E_{4}\xi^{2}\left(36\frac{dp}{dz}\left(r^{2}-3R^{2}\right)-\left(E_{3}\gamma -E_{2}\xi^{2}\right)\left(r^{4}-8r^{2}R^{2}+19R^{4}\right)\right)\\ & +E_{1}^{2}\left\{\begin{array}{l} 576E_{4}\beta \left(-6\left(r^{2}+R^{2}\right)+\left(r^{4}+r^{2}R^{2}+R^{4}\right)\beta \right)+\beta_{m}E_{3}\gamma \\ \left(16\left(r^{4}+r^{2}R^{2}-8R^{4}\right)-\left(r^{6}+r^{4}R^{2}+r^{2}R^{4}-15R^{6}\right)\beta \right) \end{array}\right\}\\ & +E_{1}\left\{\begin{array}{l} E_{3}\gamma \left(\begin{array}{l} \beta_{m}E_{2}\xi^{2}\left(r^{6}-7r^{4}R^{2}+29r^{2}R^{4}-59R^{6}\right)\\ -288E_{4}\left(6R^{2}+r^{4}\beta -2R^{4}\beta -2r^{2}\left(1+R^{2}\beta \right)\right) \end{array}\right)\\ +32E_{4}\left(108\frac{dp}{dz}\left(r^{2}+R^{2}\right)\beta +E_{2}\xi^{2}\left(\begin{array}{l} 54R^{2}+10r^{4}\beta -26R^{4}\beta \\ -r^{2}\left(18+17R^{2}\beta \right) \end{array}\right)\right) \end{array}\right\}, \end{array}$$

(26) $$\begin{array}{ll} {\bar{\theta }}_{2} & =\frac{\beta_{m}E_{2}\xi^{2}\left(r^{2}-R^{2}\right)}{73728E_{1}E_{4}}\\ & \times \left[\begin{array}{l} 128\frac{dp}{dz}\left(2r^{4}-7r^{2}R^{2}+11R^{4}\right)-\left(E_{3}\gamma -E_{2}\xi^{2}\right)\left(9r^{6}-71r^{4}R^{2}+181r^{2}R^{4}-251R^{6}\right)\\ +2E_{1}\left\{\begin{array}{l} 9r^{6}\beta +r^{2}R^{2}\left(448-43R^{2}\beta \right)\\ -r^{4}\left(128+7R^{2}\beta \right)+R^{4}\left(-704+101R^{2}\beta \right) \end{array}\right\} \end{array}\right], \end{array}$$

The approximate series solutions can be written as

(27) $$w=\underset{\varepsilon \to 1}{\lim}\bar{u}={\bar{u}}_{0}+\varepsilon {\bar{u}}_{1}+\varepsilon {\bar{u}}_{2}+\ldots ,$$

(28) $$\theta =\underset{\varepsilon \to 1}{\lim}\bar{\theta }={\bar{\theta }}_{0}+\varepsilon {\bar{\theta }}_{1}+\varepsilon {\bar{\theta }}_{2}+\ldots ,$$

The flux (*Q*) can be computed with the help of following expression

(29) $$Q=2\overset{R}{\underset{0}{\int }}ru\left(r,z\right)dr.$$

The impedance expression may be determined using the preceding Equation (29), thus we obtain

(30) $$I_{m}=\frac{1}{Q}\int_{0}^{L}\left(-\frac{dp}{dz}\right)dz.$$

## 4. Discussion

This section is dedicated to the discussion of the graphical plots of results for all the parameters appearing in the governing equations. Particularly, the results are evaluated for velocity mechanism, temperature profile, impedance profile, wall shear stress, and trapping mechanism. These results are presented for three different cases including converging tapered artery, diverging tapered artery and non-tapered artery (see Figure 1 and its description in Section 2). The numerical values used for thermo-physical computations are given in Table 2.

### 4.1. Velocity Mechanism−w

**Figure 2:**
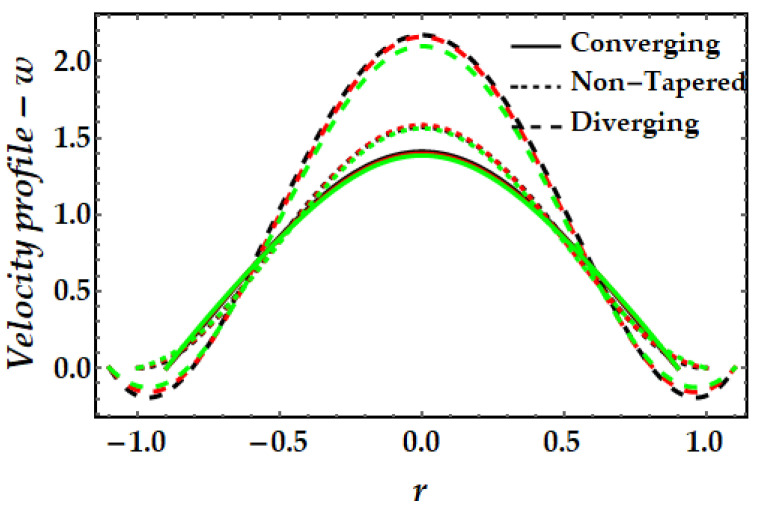
Velocity mechanism for multitudinous values of β. Black line is for β=1, Red line is for β=3, Green line is for β=4.

**Figure 3:**
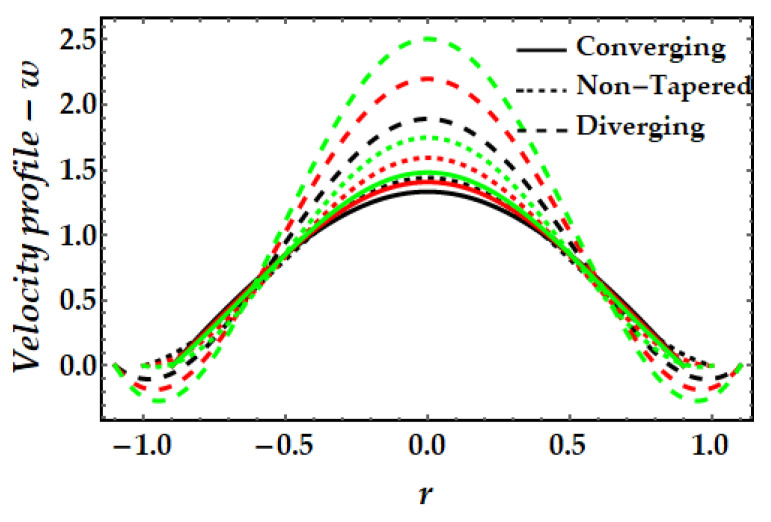
Velocity mechanism for multitudinous values of γ. Black line is for γ=2, Red line is for γ=2.5, Green line is for γ = 3.

**Figure 4:**
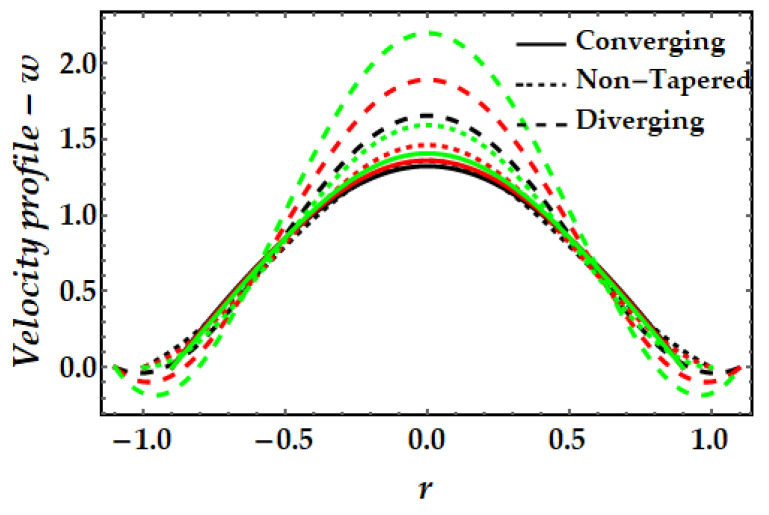
Velocity mechanism for multitudinous values of ξ. Black line is for ξ=1, Red line is for ξ=1.2, Green line is for ξ=1.4.

**Figure 5:**
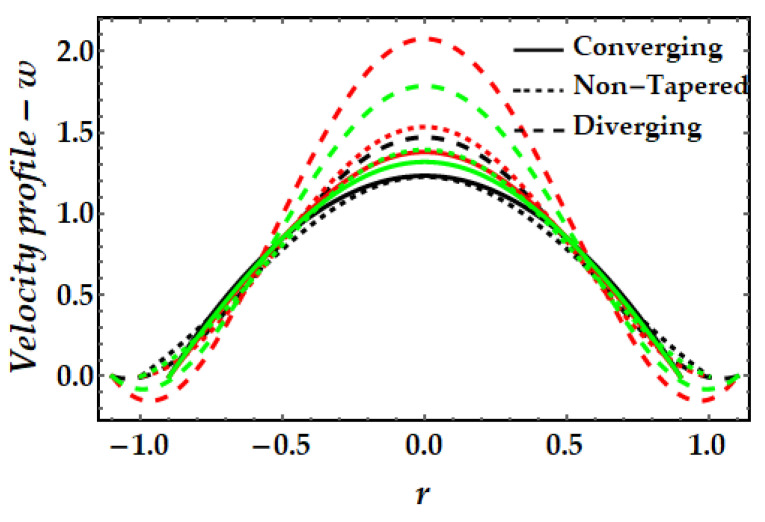
Velocity mechanism for multitudinous values of $\phi_{\mathrm{Gold}},\;\phi_{\mathrm{Copper}}$. Black line is for $\phi_{\mathrm{Gold}}=0,\;\phi_{\mathrm{Copper}}=0.1$, Red line is for $\phi_{\mathrm{Gold}}=0.1,\;\phi_{\mathrm{Copper}}=0$, Green line is for $\phi_{\mathrm{Gold}}=0.3,\;\phi_{\mathrm{Copper}}=0.3$.

Figure 2, Figure 3, Figure 4 and Figure 5 represents the variation of velocity distribution against fluid parameter β, thermal Grashof number γ, magnetic parameter ξ, and nanoparticle volume fraction $\phi_{\mathrm{Gold}},\phi_{\mathrm{Copper}}$. From Figure 2, it is seen that for higher values of the fluid parameter, the velocity profile exhibits insignificant diminishing tendency. The fluid’s velocity is higher at the center of the channel. The velocity field likewise rises when the converging artery transforms into non-tapered or divergent configurations. Furthermore, when β=0 (see Equation (7)), the current findings exhibit Newtonian fluid behavior. Figure 3 depicts the impact of the thermal Grashof number on the velocity profile. As can be seen, the velocity profile dramatically increases as the thermal Grashof number grows. Due to the growth in the thermal Grashof number, the buoyancy forces +γθ in Equation (7) also get boosted. This strengthens the natural convection currents and elevates the velocity’s magnitude. Again, the velocity intensity of diverging situations is greater than that of converging cases. The external magnetic field implications on the velocity profile are demonstrated in Figure 4. Here, it is clear that the velocity profile exhibits dual behavior over the whole domain; for instance, when r∈(−0.5,0.5), the velocity profile exhibits a rising mechanism, whilst in the remaining regions, a decreasing mechanism is seen. The fluctuation in nanoparticle volume fraction for gold and copper is illustrated in Figure 5. Here, the black and red lines indicate the mono nanofluid, with the black line indicating the absence of gold nanoparticles and the red line indicating the absence of copper nanoparticles. It is observed that gold nanoparticles have a greater impact on the magnitude of the velocity than copper nanoparticles. Again, the velocity is at its peak near the center of the artery. However, nearer to the artery walls, its effect is weaker.

### 4.2. Temperature Mechanism−θ

**Figure 6:**
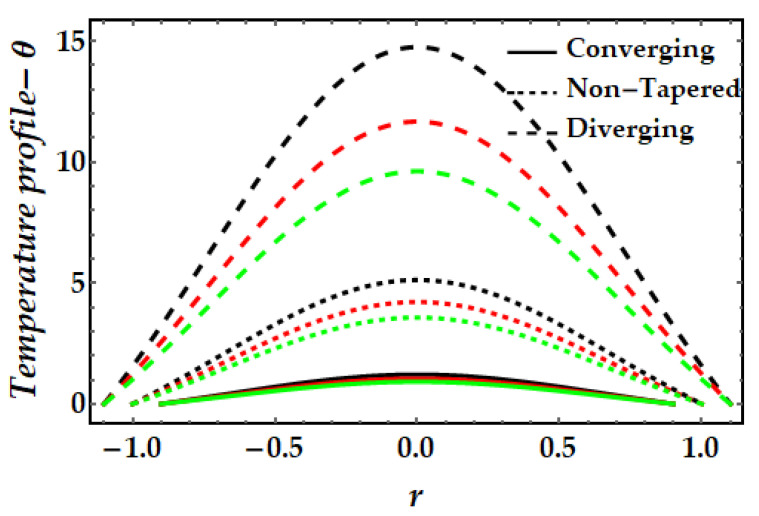
Temperature mechanism for multitudinous values of β. Black line is for β=1, Red line is for β=1.5, Green line is for β=2.

**Figure 7:**
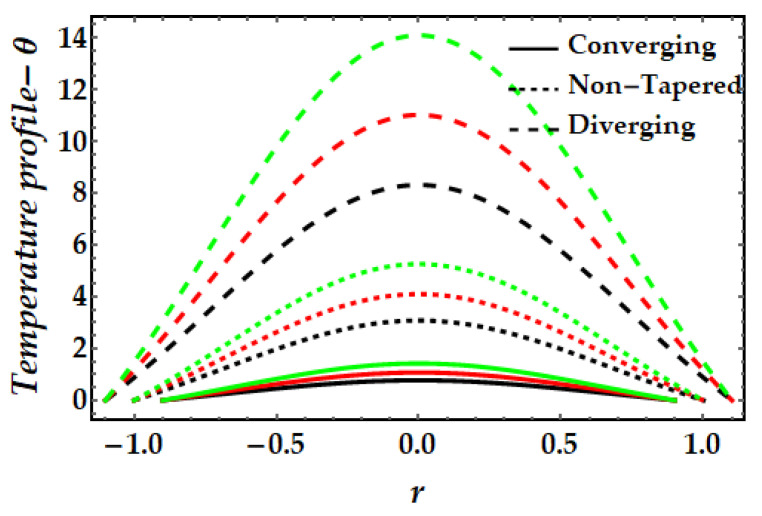
Temperature mechanism for multitudinous values of $\beta_{m}$. Black line is for $\beta_{m}=11$, Red line is for $\beta_{m}=13$, Green line is for $\beta_{m}=15$.

**Figure 8:**
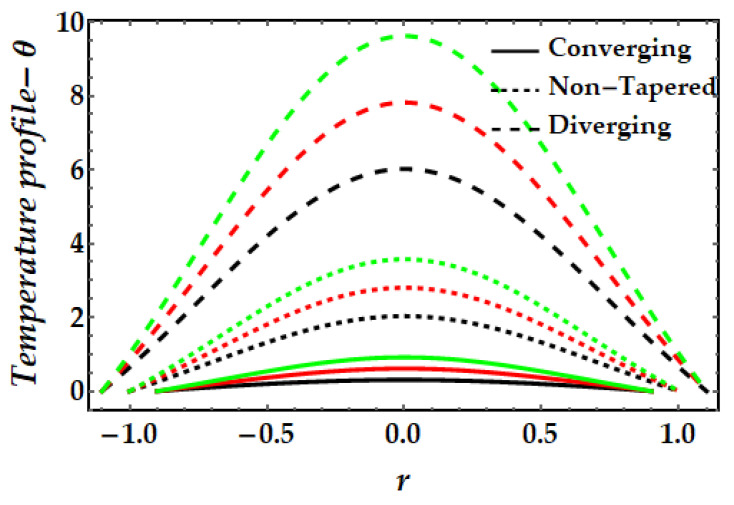
Temperature mechanism for multitudinous values of γ. Black line is for γ=2, Red line is for γ=2.5, Green line is for γ=3.

**Figure 9:**
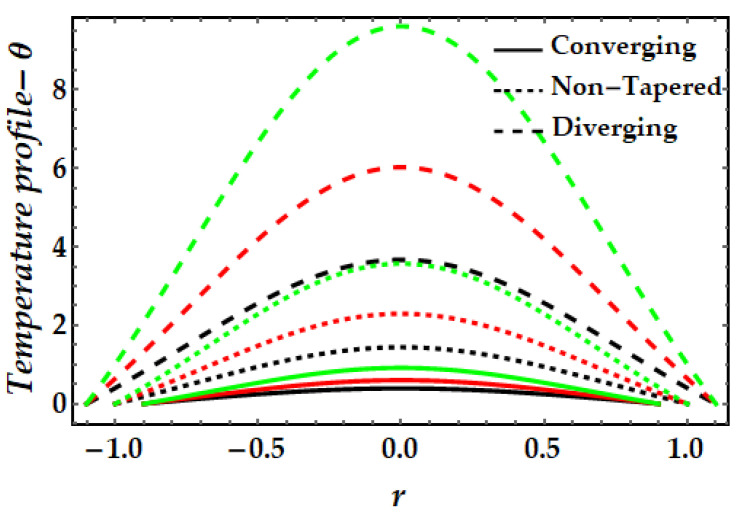
Temperature mechanism for multitudinous values of ξ. Black line is for ξ=1, Red line is for ξ=1.2, Green line is for γ=1.4.

**Figure 10:**
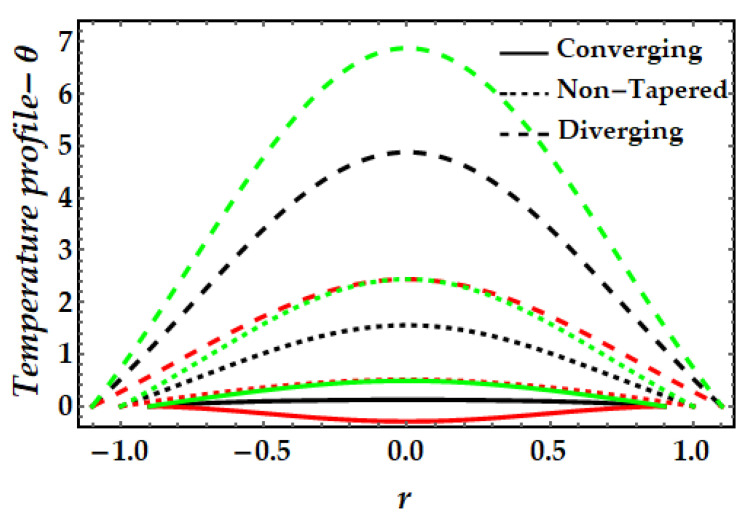
Temperature mechanism for multitudinous values of $\phi_{\mathrm{Gold}},\;\phi_{\mathrm{Copper}}$. Black line is for $\phi_{\mathrm{Gold}}=0.05,\;\phi_{\mathrm{Copper}}=0$, Red line is for $\phi_{\mathrm{Gold}}=0,\;\phi_{\mathrm{Copper}}=0.05$, Green line is for $\phi_{\mathrm{Gold}}=0.3,\;\phi_{\mathrm{Copper}}=0.3$.

The thermal profile variation for each of the emerging elements is shown in Figure 6, Figure 7, Figure 8, Figure 9 and Figure 10. In Figure 6 it can be seen that when the fluid parameter β rises, the temperature profile declines. Moreover, the temperature profile for the converging artery has the smallest magnitude, whereas the diverging artery has the highest temperature. Figure 7 demonstrates that the Brinkman number $\beta_{m}$ considerably increases the temperature profile for all arterial arrangements. Due to the fact that a greater value of Brinkman number inhibits the heat conduction caused by viscous dissipation, the temperature profile increases. Figure 8 indicates the impact of the thermal Grashof number on the temperature field. The thermal Grashof number rises the temperature distribution in comparison to all geometric shapes. Figure 9 demonstrates that a strong magnetic field effect boosts the temperature field. Due to the existence of $+\beta_{m}\xi^{2}$ in Equation (7), commonly known as Joule dissipation (or ohmic heating), this process happens. The additional effort required to pull the working fluid against the activity of an extrinsic magnetic field is wasted as thermal energy. This boosts the temperature field and heats the working fluid. Figure 10 demonstrates the impact of temperature on the nanoparticle volume fraction of gold and copper. In this illustration, the black and red lines indicate the variation of mono nanofluids, whereas the green line illustrates the behavior of hybrid nanoparticles. This graph demonstrates that the presence of copper nanoparticles (when $\phi_{\mathrm{Gold}}=0,\;\phi_{\mathrm{Copper}}=0.05$) lowers the temperature profile more than that of gold nanoparticles (when $\phi_{\mathrm{Gold}}=0.05,\;\phi_{\mathrm{Copper}}=0$). For hybrid nanofluids, the magnitude of the temperature field has been significantly raised.

### 4.3. Impedance Profile−I_m_

**Figure 11:**
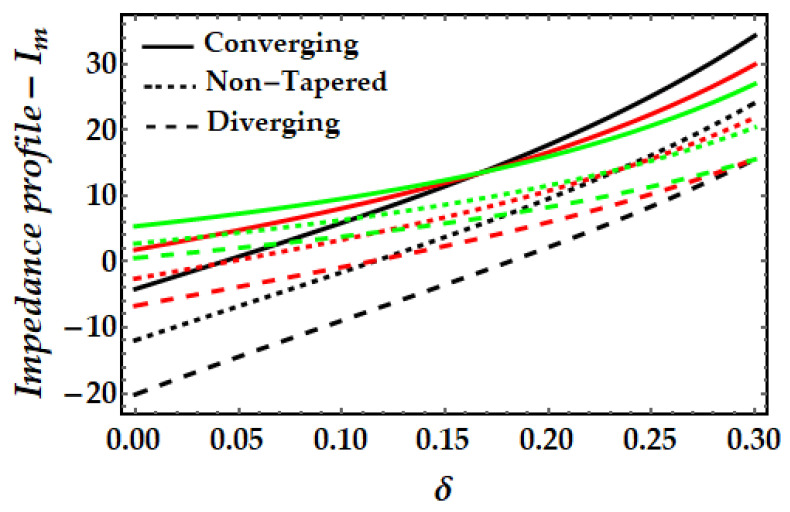
Impedance profile for multitudinous values of β. Black line is for β=1, Red line is for β=2, Green line is for β=3.

**Figure 12:**
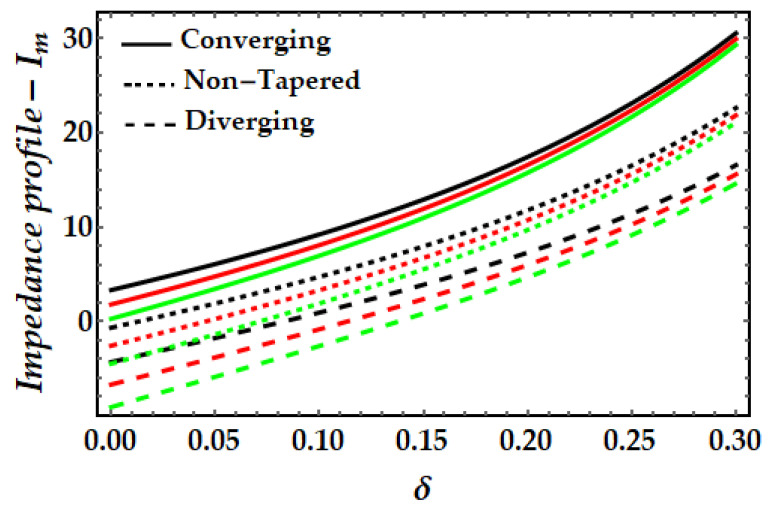
Impedance profile for multitudinous values of γ. Black line is for γ=2, Red line is for γ=2.5, Green line is for γ=3.

**Figure 13:**
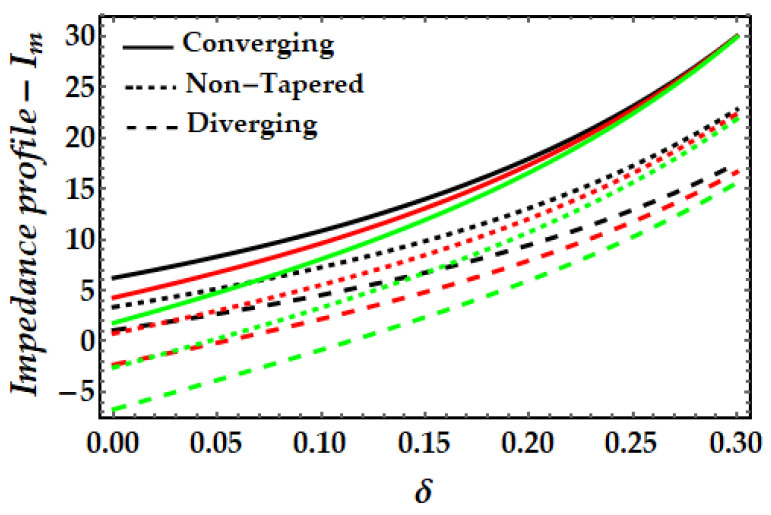
Impedance profile for multitudinous values of ξ. Black line is for ξ=1, Red line is for ξ=1.2, Green line is for γ=1.4.

**Figure 14:**
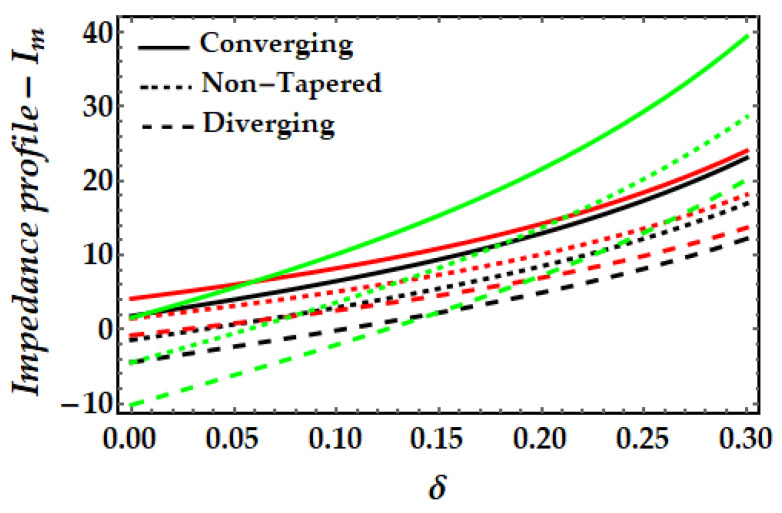
Impedance profile for multitudinous values of $\phi_{\mathrm{Gold}},\;\phi_{\mathrm{Copper}}$. Black line is for $\phi_{\mathrm{Gold}}=0.1,\;\phi_{\mathrm{Copper}}=0$, Red line is for $\phi_{\mathrm{Gold}}=0,\;\phi_{\mathrm{Copper}}=0.1$, Green line is for $\phi_{\mathrm{Gold}}=0.15,\;\phi_{\mathrm{Copper}}=0.15$.

Figure 11, Figure 12, Figure 13 and Figure 14 depict the impedance curve with stenosis height for different values of all significant parameters. It is evident here that the impedance profile exhibits diverse behavior for various fluid parameter values. There are three critical points, which as δ=0.16,0.24,0.3; before these points, the impedance profile exhibits a rising trend; however, after these points, the impedance profile exhibits a decreasing trend. In circumstances of convergence, the maximum magnitude of the impedance profile occurs. Figure 12 depicts that when the thermal Grashof number rises, the impedance profile for all three arterial conditions demonstrates a remarkable resistance. The effect of magnetic field on the impedance profile is predicted in Figure 13. Here, we can also see that the magnetic field depletes the formation of the impedance profile and exhibits a diminishing trend in all geometrical configuration. Figure 14 depicts the behavior of mono nanofluid and hybrid nanofluid, and it is discovered that the impedance profile has a maximum magnitude when both nanoparticles are present. In contrast, the impedance profile exhibits a declining trend in all three artery conditions (convergent, non-tapered, and divergent).

### 4.4. Wall Shear Stress−S_rz_

**Figure 15:**
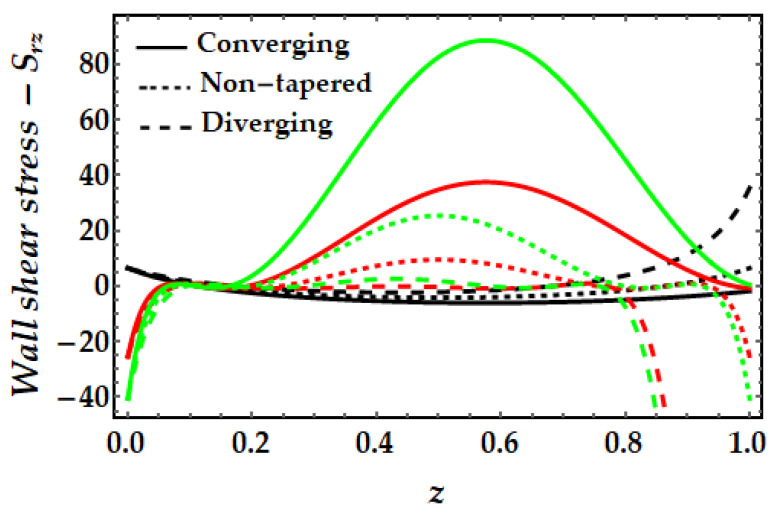
Wall shear stress for multitudinous values of β. Black line is for β=1, Red line is for β=1.5, Green line is for β=2.

**Figure 16:**
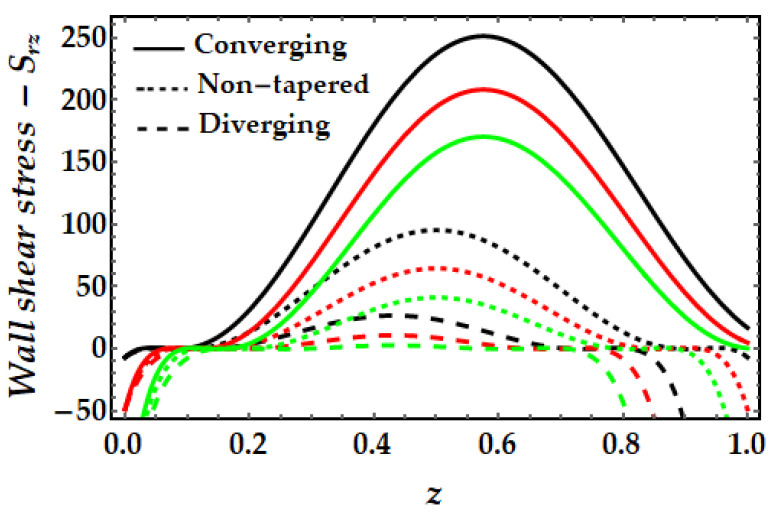
Wall shear stress for multitudinous values of γ. Black line is for γ = 2, Red line is for γ = 2.5, Green line is for γ=3.

**Figure 17:**
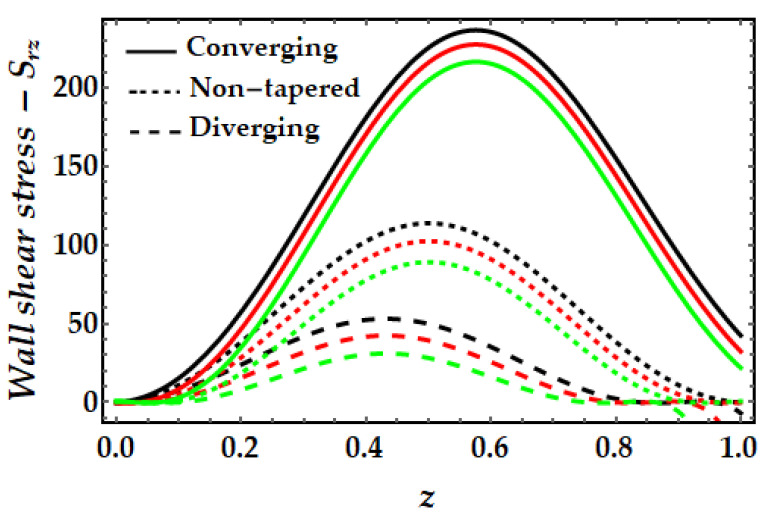
Wall shear stress for multitudinous values of ξ. Black line is for ξ=1, Red line is for ξ=1.2, Green line is for ξ=1.4.

**Figure 18:**
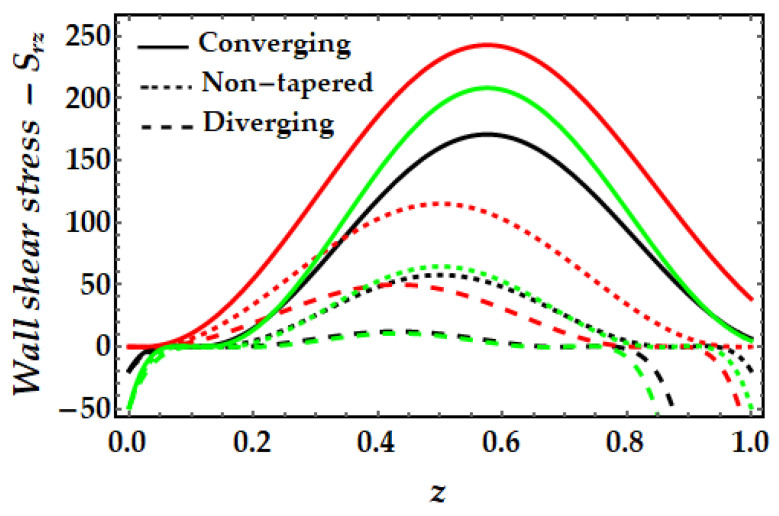
Wall shear stress for multitudinous values of $\phi_{\mathrm{Gold}},\;\phi_{\mathrm{Copper}}$. Black line is for $\phi_{\mathrm{Gold}}=0.1,\;\phi_{\mathrm{Copper}}=0$, Red line is for $\phi_{\mathrm{Gold}}=0,\;\phi_{\mathrm{Copper}}=0.1$, Green line is for $\phi_{\mathrm{Gold}}=0.1,\;\phi_{\mathrm{Copper}}=0.1$.

Figure 15, Figure 16, Figure 17 and Figure 18 depict the fluctuation of wall shear stress for all emergent parameters in the dominating mathematical simulation. Wall shear stress in blood flow is significant in the pathogenesis of atherosclerosis. Figure 15 indicates that when the Sutterby fluid parameter rises, the wall shear stress increases. We may also see some negative shear stress occurring at certain spots, which might suggest reversed flow. This mechanism occurs when the boundary layer separates from the surface. Figure 16 illustrates how the thermal Grashof number tends to resist the emergence of wall shear stress and demonstrates the declining process. However, it was observed that the existence of a magnetic field also opposes the wall shear stress as shown in Figure 17. Furthermore, we can observe that when artery shapes shift from converging to non-tapered and eventually diverging, wall shear stress decreases. The last Figure 18 demonstrates that the wall shear stress is strongest for the mono nanofluid $\left(\phi_{\mathrm{Gold}}=0\right)$ and smallest when copper nanoparticles are excluded $\left(\phi_{\mathrm{Copper}}=0\right)$. It demonstrates that the presence of nanoparticles has a substantial impact on the wall shear stress and may generate negative shear stress at specific spots.

### 4.5. Trapping Mechanism

**Figure 19:**
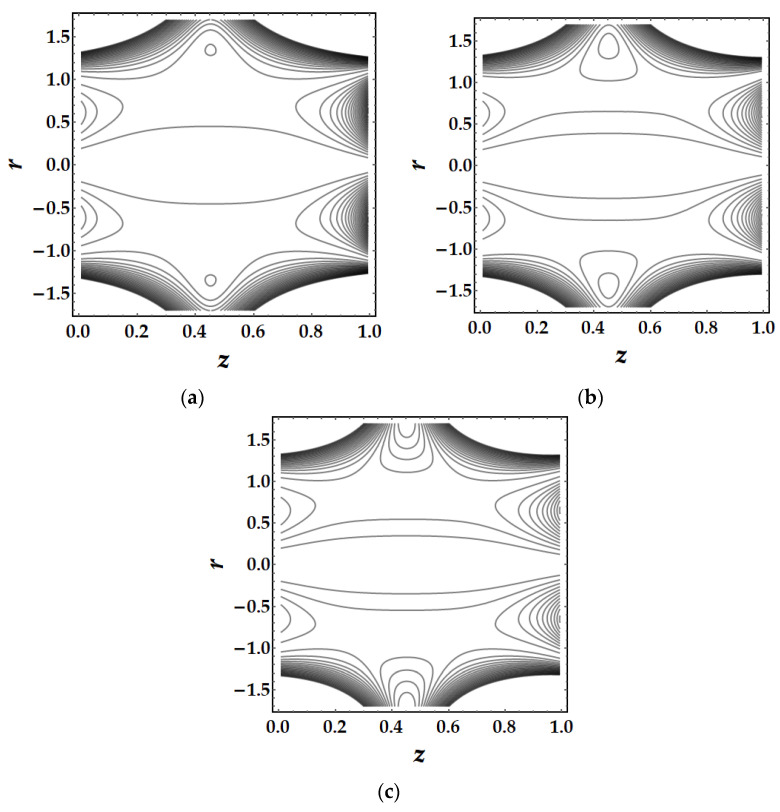
Streamlines for multitudinous values of β. (**a**) β=1.7, (**b**) β=2, (**c**) β=2.5.

**Figure 20:**
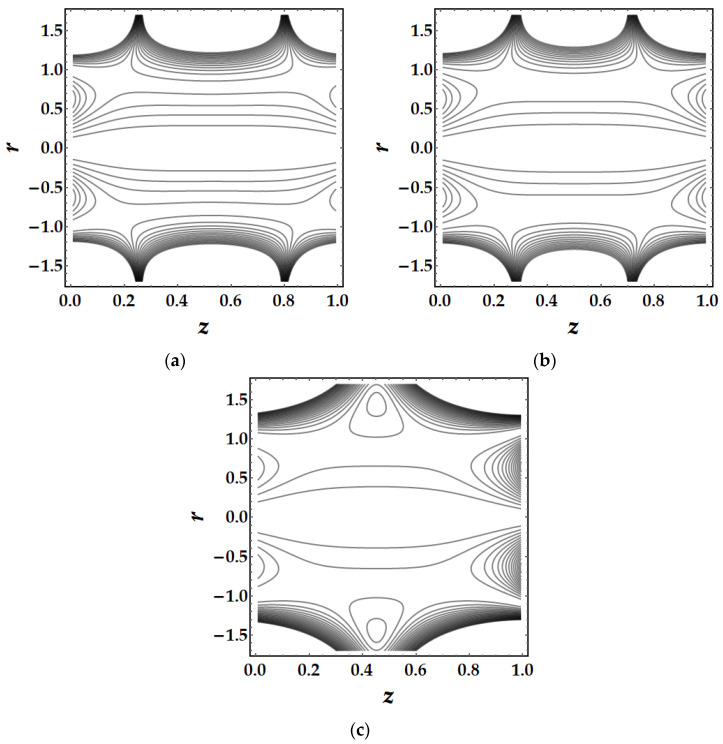
Streamlines for multitudinous values of ψ. (**a**) ψ=−0.1, (**b**) ψ=0, (**c**) ψ=0.1.

**Figure 21:**
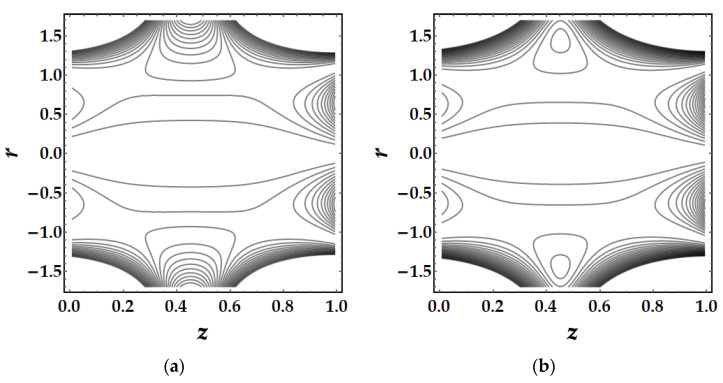

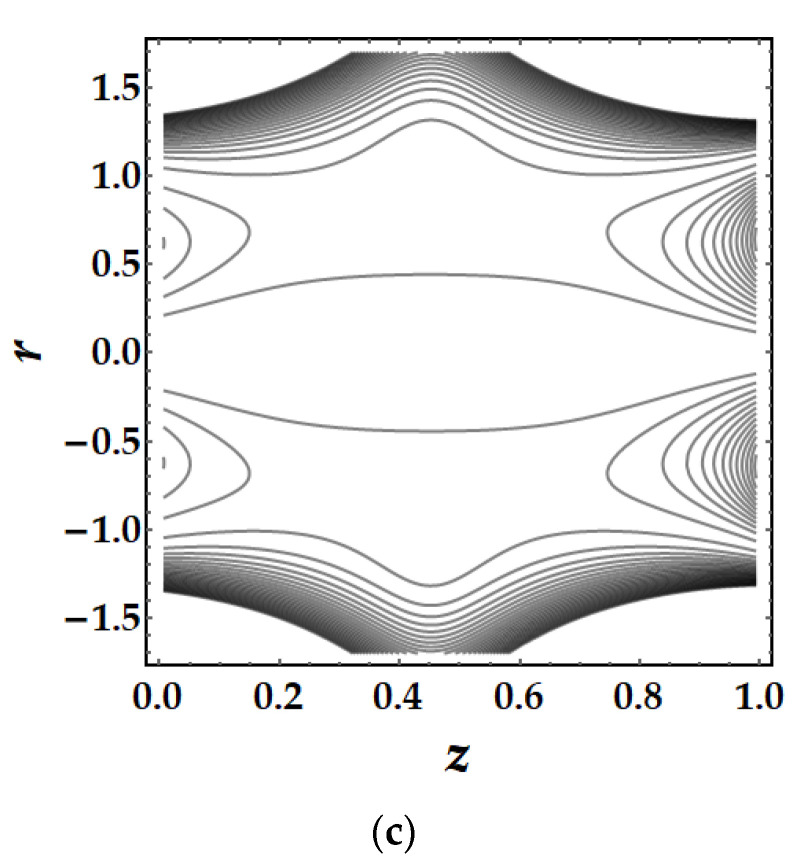
Streamlines for multitudinous values of γ. (**a**) γ=1.8, (**b**) γ=2, (**c**) γ=2.2.

**Figure 22:**
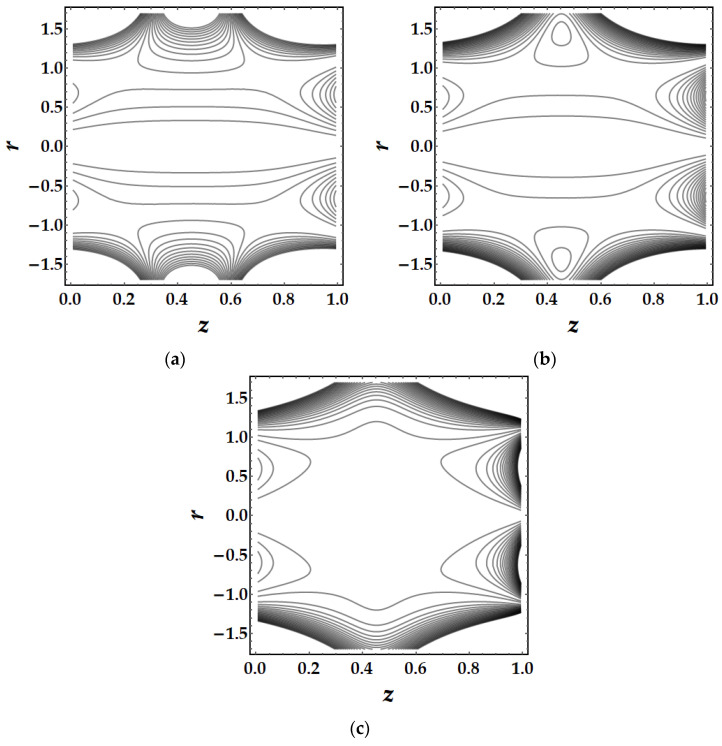
Streamlines for multitudinous values of ξ. (**a**) ξ=2.7, (**b**) ξ=3, (**c**) ξ=3.2.

**Figure 23:**
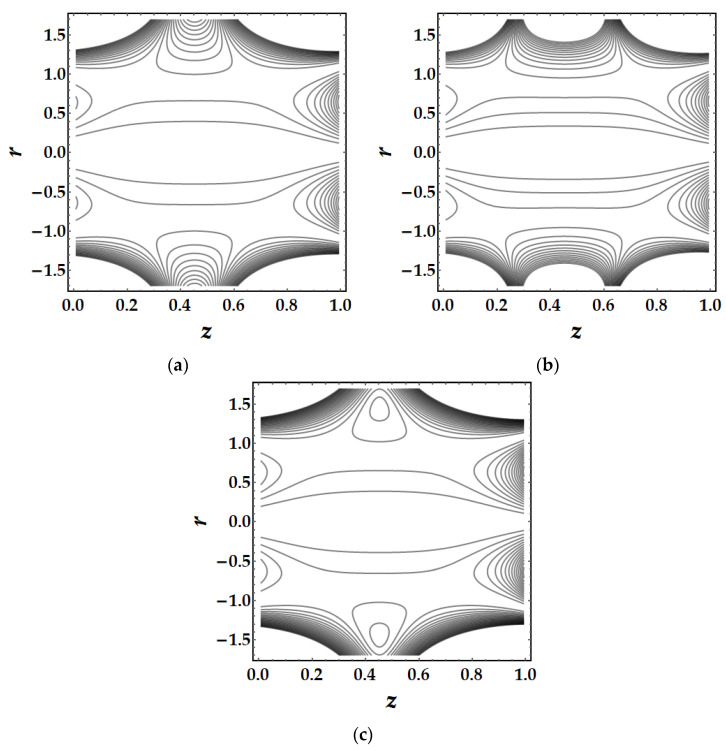
Streamlines for multitudinous values of $\phi_{\mathrm{Gold}},\;\phi_{\mathrm{Copper}}$. (**a**) $\phi_{\mathrm{Gold}}=0.1,\;\phi_{\mathrm{Copper}}=0$, (**b**) $\phi_{\mathrm{Gold}}=0,\;\phi_{\mathrm{Copper}}=0.2$, (**c**) $\phi_{\mathrm{Gold}}=0.1,\;\phi_{\mathrm{Copper}}=0.1$.

Trapping is a major influencing factor in the flow of nanofluids that can be examined by observing the trajectories. Trapping is the creation of internally moving free eddies surrounded by streamlines in the blood. This mechanism is very significant in biology since it assists in the production of blood clots and the spread of pathogens. Figure 19 demonstrates the impact of Sutterby fluid parameter fluctuation on trapping phenomena. It can be seen that the effect of fluid parameter not only affects the size of the trapping boluses, but also causes the bolus to vanish as the fluid parameter strength increases. Figure 20 depicts the consequences of tapering angle fluctuations on trapping phenomena. It can be observed that no bolus occurs in converging and non-tapered arteries, however a bolus appears in diverging arteries. Figure 21 shows that the thermal Grashof number has a significant influence on the trapping phenomenon. For example, the trapping boluses emerge for only certain values of the thermal Grashof number (see Figure 21b) but vanish for the other values. In Figure 22, a similar behavioral pattern is seen for various Hartmann number values. Figure 23 illustrates that mono nanofluid does not have a circulating bolus in the working fluid, but when hybrid nanofluid is present, a circulating bolus occurs in the fluid.

## 5. Conclusions

In this paper, we analyzed the hemodynamics of hybrid nanofluid flow, with a specific emphasis on magnetic drug targeting. In the presence of gold and copper nanoparticles, flow is considered to be propagating in the tapering stenosed artery. Because blood acts as non-Newtonian in nature, the non-Newtonian Sutterby fluid model is supposed to be a base fluid. To comprehend the rheology of the hybrid nanofluid flow, several impacts, such as magnetic field, Joule heating, and viscous dissipation, are taken into consideration in the mathematical modeling. To model and solve the stipulated nonlinear differential equations, the lubrication technique and perturbation method are utilized. The primary findings of the research context are outlined below:

(i) The Sutterby fluid parameter opposes the flow negligibly, whereas the Hartmann number and thermal Grashof number strengthen the flow field. In addition, the thermal Grashof number and the Hartmann number exhibit a decreasing tendency closer to the walls.
(ii) It is also observed that the addition of gold nanoparticles (mono nanofluids) results in a greater magnitude of velocity than copper nanofluids.
(iii) The thermal profile exhibits a diminishing trend owing to greater values of the Sutterby fluid parameter, whereas a rising trend is detected due to the significant impact of the magnetic field, Brinkman number, and thermal Grashof number.
(iv) Copper nanoparticles (in the absence of gold nanoparticles) are observed to deplete the thermal profile substantially more than gold nanoparticles. Nevertheless, the thermal profile is enhanced by the presence of both nanoparticles (hybrid nanofluids).
(v) It is observed that the impedance profile has a dual pattern for various values of the Sutterby fluid parameter, but the thermal Grashof number and magnetic field exhibit a uniformly declining tendency.
(vi) When the impact of both nanoparticles rises, the impedance profile grows while the amplitude of the impedance profile for mono nanofluids decreases.
(vii) For greater values of the Sutterby fluid parameter, the wall shear stress has been observed to rise considerably, whereas the inverse is true for the Hartmann number and the thermal Grashof number.
(viii) The trapping mechanism demonstrates that the fluid parameters influence the size and frequency of the bolus. However, for other parameters, the trapped bolus manifested for certain values, although for converging and non-tapered arteries, it did not.

## Figures and Tables

**Table 1:** Thermal & physical properties of nanofluid and hybrid nanofluid.

	Nanofluid	Hybrid Nanofluid
Dynamic viscosity	$\mu_{\;nf}=\frac{\mu_{\mathrm{Blood}}}{{\left(1-\phi_{\mathrm{Gold}}\right)}^{2.5}}$	$\mu_{\;hnf}=\frac{\mu_{nf}}{{\left(1-\phi_{\;\mathrm{Copper}}\right)}^{2.5}}$
Density	$\rho_{nf}=\;\left(1-\phi_{\;\mathrm{Gold}}\right)\rho_{\mathrm{Blood}}+\rho_{\mathrm{Gold}}\phi_{\;\mathrm{Gold}}$	$\rho_{hnf}=\left(1-\phi_{\;\mathrm{Copper}}\right)\rho_{nf}+\;\rho_{\mathrm{copper}}\phi_{\;\mathrm{Copper}}$
Electrical conductivity	$W_{nf}=W_{f}\left[\frac{W_{\mathrm{Gold}}\left(1+2f_{\mathrm{Gold}}\right)+2W_{\mathrm{Blood}}\left(1-2f_{\mathrm{Gold}}\right)}{W_{\mathrm{Gold}}\left(1-f_{\mathrm{Gold}}\right)+W_{\mathrm{Blood}}\left(2+f_{\mathrm{Gold}}\right)}\right]$	$W_{hnf}=W_{nf}\left[\frac{W_{\mathrm{Copper}}\left(1+2f_{\mathrm{Copper}}\right)+2W_{\mathrm{Blood}}\left(1-2f_{\mathrm{Copper}}\right)}{W_{\mathrm{Copper}}\left(1-f_{\mathrm{Copper}}\right)+W_{\mathrm{Blood}}\left(2+f_{\mathrm{Copper}}\right)}\right]$
Thermal conductivity	$k_{nf}=k_{f}\left[\frac{2k_{\mathrm{Blood}}+k_{\mathrm{Gold}}-2\left(k_{\mathrm{Blood}}-k_{\mathrm{Gold}}\right)\;f_{\mathrm{Gold}}}{2k_{\mathrm{Blood}}+k_{\mathrm{Gold}}+\left(k_{\mathrm{Blood}}-k_{\mathrm{Gold}}\right)\;f_{\mathrm{Gold}}}\right]$	$k_{hnf}=k_{nf}\left[\frac{2k_{f}+k_{\mathrm{Copper}}-2\left(k_{f}-k_{\mathrm{Copper}}\right)\;f_{\mathrm{Copper}}}{2k_{f}+k_{\mathrm{Copper}}+\left(k_{f}-k_{\mathrm{Copper}}\right)\;f_{\mathrm{Copper}}}\right]$
Heat capacity	${\left(\rho C_{p}\right)}_{n\;f}=(1-\phi_{\;\mathrm{Gold}})\;{\left(\rho \;C_{p}\right)}_{\mathrm{Blood}}+\phi_{\;\mathrm{Gold}}{\left(\rho \;C_{p}\right)}_{\mathrm{Gold}}$	${\left(\rho C_{p}\right)}_{hn\;f}=(1-\phi_{\mathrm{Copper}})\;{\left(\rho \;C_{p}\right)}_{nf}+\phi_{\mathrm{Copper}}{\left(\rho C_{p}\right)}_{\mathrm{copper}}$
Thermal expansion	${\left(rb\right)}_{nf}=\left(1-f_{\mathrm{Gold}}\right)\;{\left(rb\right)}_{\mathrm{Blood}}+f_{\mathrm{Gold}}{\left(rb\right)}_{\mathrm{Gold}}$	${\left(rb\right)}_{hnf}=\left(1-f_{\mathrm{Copper}}\right)\;{\left(rb\right)}_{nf}+f_{\mathrm{Copper}}{\left(rb\right)}_{\mathrm{Copper}}$

**Table 2:** Computational values of Thermal & physical properties of hybrid nanofluid used for computational results [52].

Physical Characteristics	Base Fluid (Blood)	Copper Nanoparticles	Gold Nanoparticles
$C_{p}\left[J/\mathrm{Kg}\cdot K\right]$	3617	385	129.1
$\rho \left[\mathrm{Kg}/m^{3}\right]$	1050	8933	19300
σ[S/m]	1.33	$5.96\times 10^{7}$	$4.5\times 10^{7}$
β[1/K]	0.18	16.65 × 10^−6^	0.0000142
k[W/m⋅K]	0.52	400	320

## Data Availability

Data is contained within the article.
